# BIOMARKERS OF SENESCENT CELL BURDEN

**DOI:** 10.1093/geroni/igz038.2048

**Published:** 2019-11-08

**Authors:** Nathan LeBrasseur

**Affiliations:** Robert and Arlene Kogod Center on Aging, Mayo Clinic, Rochester, Minnesota, United States

## Abstract

Senescent cells drive aging. Preclinical studies suggest that targeted elimination of senescent cells offers a unique therapeutic approach to counter numerous chronic diseases and geriatric syndromes. To foster the translation of basic science discoveries to clinical application, we have sought to identify circulating biomarkers that reflect systemic senescent cell burden. We first analyzed the secretome of multiple senescent human cell-types and developed a candidate panel of proteins that could be reliably measured in human blood. Multiple proteins demonstrated significant associations with chronological age in a community-based cohort of adults aged 20-to-90 years. Impressively, in two distinct surgical cohorts (severe aortic stenosis and ovarian cancer), candidate protein concentrations were associated with biological age indices, including frailty and adverse outcomes. Our data suggest senescence biomarkers may have utility for clinical practice as indicators of risk, and for clinical research as surrogate endpoints in trials of interventions targeting senescent cells.

